# Fingerprinting Interactions between Proteins and Ligands for Facilitating Machine Learning in Drug Discovery

**DOI:** 10.3390/biom14010072

**Published:** 2024-01-05

**Authors:** Zoe Li, Ruili Huang, Menghang Xia, Tucker A. Patterson, Huixiao Hong

**Affiliations:** 1 National Center for Toxicological Research, US Food and Drug Administration, Jefferson, AR 72079, USA; zoe.li@fda.hhs.gov (Z.L.); tucker.patterson@fda.hhs.gov (T.A.P.); 2 National Center for Advancing Translational Sciences, National Institutes of Health, Bethesda, MD 20892, USA; ruili.huang@nih.gov (R.H.); mxia@mail.nih.gov (M.X.)

**Keywords:** molecular fingerprints, 3D structural interaction fingerprints, machine learning, drug discovery, structure–activity relationships, protein–ligand interactions, predictive modeling

## Abstract

Molecular recognition is fundamental in biology, underpinning intricate processes through specific protein–ligand interactions. This understanding is pivotal in drug discovery, yet traditional experimental methods face limitations in exploring the vast chemical space. Computational approaches, notably quantitative structure–activity/property relationship analysis, have gained prominence. Molecular fingerprints encode molecular structures and serve as property profiles, which are essential in drug discovery. While two-dimensional (2D) fingerprints are commonly used, three-dimensional (3D) structural interaction fingerprints offer enhanced structural features specific to target proteins. Machine learning models trained on interaction fingerprints enable precise binding prediction. Recent focus has shifted to structure-based predictive modeling, with machine-learning scoring functions excelling due to feature engineering guided by key interactions. Notably, 3D interaction fingerprints are gaining ground due to their robustness. Various structural interaction fingerprints have been developed and used in drug discovery, each with unique capabilities. This review recapitulates the developed structural interaction fingerprints and provides two case studies to illustrate the power of interaction fingerprint-driven machine learning. The first elucidates structure–activity relationships in β2 adrenoceptor ligands, demonstrating the ability to differentiate agonists and antagonists. The second employs a retrosynthesis-based pre-trained molecular representation to predict protein–ligand dissociation rates, offering insights into binding kinetics. Despite remarkable progress, challenges persist in interpreting complex machine learning models built on 3D fingerprints, emphasizing the need for strategies to make predictions interpretable. Binding site plasticity and induced fit effects pose additional complexities. Interaction fingerprints are promising but require continued research to harness their full potential.

## 1. Introduction

Molecular recognition is a fundamental process in living organisms, involving specific and high-affinity interactions between biological macromolecules and various small molecules, leading to the formation of specific complexes [1,2]. Among these macromolecules, proteins play a vital role as they carry out their functions by binding to themselves or other molecules [2]. Consequently, a comprehensive understanding of protein–ligand interactions holds the key to unraveling the intricacies of molecular biology. Additionally, this knowledge about the mechanisms governing protein–ligand recognition and binding serves as a valuable resource in drug discovery, design, and development. By delving into the specifics of these interactions, researchers can better advance their quest for new therapeutic agents and foster scientific advancements in the field of drug development.

Traditional experimental methods have long been employed to predict the binding activity of small molecules [3]. These methods include isothermal titration calorimetry, fluorescence thermal shift assay, cellular thermal shift assay, and analytical ultracentrifugation, among others [3]. However, the vastness of the chemical space allows for an astounding number of approximately 10^60^ possible small molecules to be synthesized [4]. Despite this immense potential, only a small fraction of the potential protein–ligand interactions has yet to be explored [4]. Efficiently navigating through this vast search space poses challenges for traditional experimental methods due to their inherent drawbacks: high cost, time consumption, and labor intensiveness. Consequently, the increasing demand for more efficient approaches to predict the biological activities of small molecules has driven the development of computational methods. These computational approaches serve as invaluable tools to streamline the search process, narrowing down the possibilities and enabling researchers to focus on promising targets.

**Figure 1:**
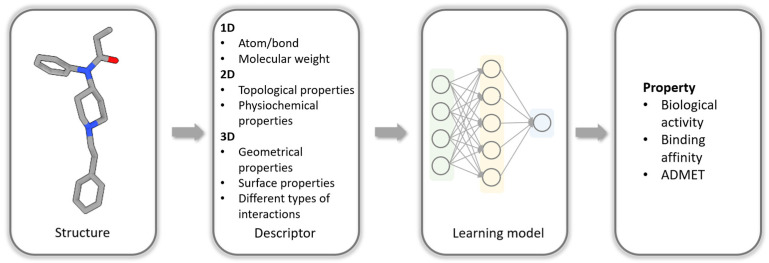
Typical workflow for using machine learning to predict properties of molecules.

One of the most widely used computational approaches in drug discovery is quantitative structure–activity/property relationship (QSAR/QSPR) analysis [5]. This approach operates on the assumption that similar molecules exhibit similar bioactivities or physicochemical properties [5,6]. Leveraging this assumption, QSAR/QSPR analysis predicts the activities or properties of new molecules by establishing correlations between their chemical or structural features and their observed activities or properties [5,6]. This approach significantly reduces the need for time-consuming and costly experimental assays. Central to QSAR/QSPR analysis is the concept of molecular similarity, which is usually measured based on various molecular descriptors and fingerprints [7,8]. Molecular descriptors are numerical descriptions of the structural features of a chemical and are widely used in the development of predictive models of predicting biological activity and chemical properties [9,10,11,12,13,14]. Fingerprints encode the structural features of a molecule. These fingerprints serve as property profiles, typically presented in the form of vectors, where each vector element represents the existence, degree, or frequency of a specific structural feature [15,16,17]. Molecular fingerprints play a fundamental role in various drug discovery processes, including virtual screening, similarity-based compound searches, target molecule ranking, drug ADMET (absorption, distribution, metabolism, excretion, and toxicity) prediction, and more. Over the past few decades, different types of two-dimensional (2D) fingerprints have been developed for molecular feature encoding [18,19,20]. These fingerprints can be extracted from molecular connection tables without requiring three-dimensional (3D) structural information. The main categories of 2D fingerprints are as follows: substructure key-based fingerprints, topological or path-based fingerprints, circular fingerprints, and pharmacophore fingerprints [21,22,23]. Two-dimensional fingerprints are advantageous due to their ease, speed, and convenience of generation, as they solely rely on 2D structures [5]. Consequently, they are extensively utilized as input for machine learning algorithms in various drug discovery applications, such as binding affinity prediction, toxicity assessment, solubility analysis, and partition coefficient estimation [24]. A typical workflow for using machine learning to predict the properties of molecules is shown in Figure 1.

In recent years, there has been a notable shift in the extensive use of machine learning from QSAR studies to focus on structure-based predictive modeling [25,26,27,28]. The availability of abundant structural and binding affinity data for protein–ligand complexes has enabled the training of binding affinity prediction models, leading to a surge in the development of machine-learning scoring functions [29]. These scoring functions exhibit exceptional performance in scoring works and have proven to outperform classical scoring functions, primarily due to their ability to handle large volumes of structural data effectively [29,30]. A critical aspect of constructing a machine-learning scoring function is feature engineering, which involves transforming complex structures into a series of descriptors. This process is guided by biologically-relevant interactions, such as hydrogen bonds, hydrophobic contacts, ionic interactions (salt bridges), π-stacking, and π-cation interactions [31].

**Figure 2:**
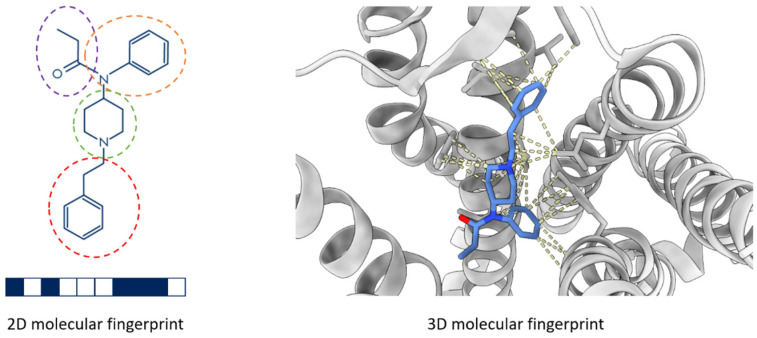
Illustration of a 2D molecular fingerprint (**left**) and a 3D molecular fingerprint (**right**). The dash circles in different colors indicate different structural features that are recorded in a bit string (under the 2D structure) as the fingerprint of the molecule. In the right sub-figure, the small molecule is represented by a stick model and the protein is drawn in a grey ribbon model. The interactions between the small molecule and the protein are indicated with yellow dashed lines and are recorded as the fingerprint of the small molecule in the protein.

Figure 2 illustrates a conventional fingerprint that is generated based only on the 2D structure of a small molecule and an emerging 3D interaction fingerprint that describes the interactions between a small molecule and its interacting macromolecule in a 3D structure. Recently, the focus of scoring function descriptors has shifted towards 3D interaction fingerprints (IFPs) because of their simplicity in representation and elaborate profiles of key interactions. IFPs are defined based on the interacting atoms between the protein and ligand within a protein–ligand complex structure. They are stored as one-dimensional (1D) vectors or matrices of Booleans, integers, or floating-point numbers, providing a concise and informative representation of the interaction patterns between the two entities [30,32]. The use of IFPs in machine-learning scoring functions holds significant promise in accurately characterizing and predicting protein–ligand interactions, thereby advancing the field of structure-based predictive modeling.

## 2. Types of Structural Interaction Fingerprints

The development and application of various structural IFPs have been significant in advancing the field of protein–ligand interaction analysis. One of the pioneering structural IFP algorithms was introduced by Deng et al. in 2004, focusing on clustering kinase–inhibitor complexes [33]. Their fingerprint encompassed seven bits per interacting amino acid, representing predefined interaction types, including backbone, sidechain, polar, hydrophobic, and H-bond donor/acceptor interactions [33]. Mordalski et al. later extended this approach by adding two bits to encode aromatic and charged interactions, leading to improved technical implementation [34]. Notably, structural IFP was instrumental in identifying the critical amino acids involved in interactions with antagonists within serotonin 5-HT7 receptor homology models [35].

Another widely used variant, developed by Marcou and Rognan in 2006, employs a seven-bit fingerprint encoding hydrophobic, aromatic face-to-face and edge-to-face, H-bond donor/acceptor, and cationic/anionic interactions [36]. Importantly, the geometric definitions in this variant can be customized, allowing for the inclusion of less common interaction types like weak H-bonds, cation-pi, and metal complexation [36]. This flexibility has enhanced the versatility of the fingerprinting approach. Later, the Rognan group devised a method to encode protein–ligand interactions into a 1D binary IFP string represented by an array of 11-bit substrings [37,38]. This novel approach effectively describes how each amino acid within the binding pocket interacts with the ligand. Specifically, every amino acid is encoded into one 11-bit substring, corresponding to 11 distinct types of interactions: hydrophobic interaction, aromatic interaction (face-to-face), aromatic interaction (edge-to-face), hydrogen bond interaction (protein atom as acceptor), hydrogen bond interaction (protein atom as donor), ionic interaction (protein atom with positive charge), ionic interaction (protein atom with negative charge), weak hydrogen bond interaction (protein atom as acceptor), weak hydrogen bond interaction (protein atom as donor), π-cation interaction, and metal ionic interaction with the ligand [37,38]. This encoding system provides a comprehensive representation of the intricate interactions between amino acids and the ligand, enabling a detailed analysis of their binding patterns.

The Rognan group also introduced triplet IFPs, where interaction points forming triangles are encoded into a fixed-length fingerprint of 210 bits [30]. The protein–ligand interaction is characterized by two interacting atoms and an interaction pseudoatom for ionic interaction, hydrogen bonding, and metal complexation. The interaction pseudoatom can be in three positions: the geometric center of the interacting atoms, near the interacting protein atom, and near the interacting ligand atom [30]. Interaction pseudoatoms can be computed using any of these three positions, allowing for mapping the interaction either on ligand atoms, protein atoms, or naturally at the mid-distance between the interacting atoms [30]. For hydrophobic interactions, when a ligand atom interacts with more than one protein atom, the interaction with the shortest distance is used to define the interaction pseudoatom. For aromatic interactions, an aromatic interaction pseudoatom is placed in the middle between the aromatic ring centroids. Although primarily designed for binding site comparison, triplet IFPs showed comparable performance to IFP in the post-processing of docking results [30].

Python-based protein–ligand interaction fingerprint (PyPLIF), an open-source Python tool developed by Radifar et al., aims at improving the accuracy of molecular docking results in virtual screening [39]. PyPLIF converts 3D interaction data from molecular docking into 1D bitstring representations, where each bit encodes the presence or absence of specific interaction types with binding site residues [39]. The similarity between these fingerprints and a reference ligand fingerprint is then evaluated using metrics like the Tanimoto coefficient [39]. Selecting top docking poses based on interaction fingerprint similarity, rather than relying solely on docking scores, significantly improves the identification of true binders [39].

Atomic pairwise interaction fingerprint (APIF) offers a binding site size-independent encoding of protein–ligand interactions. It achieves this by considering the relative position and interaction type of all pairs of interacting atoms between the ligand and protein [40]. Each interacting atom pair is categorized by its interaction type, such as the hydrophobic-acceptor, and sorted into discrete distance ranges between the ligand and protein atoms [40]. Consequently, a 294-bit fixed-length binary fingerprint is generated, encompassing various combinations of interaction pairs and distances. APIF’s utilization of relative geometry rather than absolute positions allows for a comparison of binding modes across diverse binding sites [40]. This 1D fingerprint retains essential 3D information, making it valuable for virtual screening and docking pose selection. However, one limitation is the reduced precision in capturing geometric details, which may make interpreting interactions from APIF challenging [40]. Despite this, APIF stands out for providing a concise representation of conserved interaction patterns, independent of the binding site size, although it may lack the intuitive interpretability found in residue-specific interaction fingerprints.

The simple ligand–receptor interaction descriptor (SILIRID) is an innovative fixed-length vector representation that derives from protein–ligand interaction fingerprints, serving to characterize binding sites. It condenses the interactions between ligand atoms and binding site residues into a concise 168-dimensional vector [41]. This is achieved by summing the binary fingerprint bits for identical amino acids and capturing their corresponding interaction types (such as hydrophobic, hydrogen bond donor/acceptor, etc.) [41]. SILIRID’s distinct feature lies in its ability to merge residue-specific fingerprints into a binding site-independent summary, facilitating the comparison of interactions across binding sites of varying sizes [41]. As a result, SILIRID offers a compact representation of conserved interaction patterns that find applications in tasks like binding site comparison, virtual screening, and the visualization of chemogenomic space. One limitation to consider is the reduction in per-residue details, which may limit the granularity of interpretation [41]. Overall, SILIRID excels in encoding essential interaction features within a size-independent vector, although it may not possess the same level of interpretability found in residue-specific fingerprints.

Another unique approach to structural protein–ligand interaction fingerprints (SPLIF) was proposed by Da and Kireev [42]. It was designed to describe and compare protein–ligand interactions in a manner that is independent of the binding site. Unlike other approaches, SPLIFs explicitly encode the 3D structures of interacting ligand and protein fragments, capturing the nuances of the interaction modes and implicitly considers various contacts, such as π-π stacking [42]. The generation of SPLIF involves expanding contacting ligand and protein atoms to include neighboring atoms within a defined radius [42]. These circular fragments are assigned identifiers, and their 3D coordinates are retrieved [42]. The SPLIF then encodes the matching circular fragments between a docking pose and the reference complex, assessing similarity through a normalized score based on the fraction of matched fragments [42]. The evaluation involves both 2D fragment identity and 3D structural alignment, providing a comprehensive representation of the interaction patterns. A notable advantage of SPLIFs is their implicit inclusion of diverse interaction types in the 3D structure description [42]. However, the trade-off is the loss of precise geometric details. Overall, SPLIFs offer a robust platform for the quantitative comparison of conserved interaction patterns across binding sites of varying sizes.

Recently, Wojcikowski et al. introduced the protein–ligand extended connectivity fingerprint (PLECFP) [43], based on the atomic environment concept of the extended connectivity fingerprint initially proposed by Rogers and Hahn in 2010 [18]. PLECFP captures the local atomic environments between the interacting protein and ligand molecules. Its construction involves identifying contacting atom pairs and characterizing the neighborhood surrounding each atom within a specified bond depth. These ligand and receptor environments are paired, and their hashed bit positions create the final folded fingerprint. PLECFP’s parameterization and evaluation on binding affinity prediction tasks using linear regression, random forest, and neural network models showcased its impressive descriptive capabilities. Surprisingly, the simple linear model performed similar with more complex methods, underscoring the richness of PLECFP’s representation. Notably, PLECFP outperformed other interaction fingerprints like SILIRID and SPLIF, yielding Pearson correlation coefficients exceeding 0.8 on benchmark datasets [43]. Such exceptional performance suggests PLECFP’s potential for diverse drug discovery tasks, including lead optimization and scaffold hopping, thanks to its implicit capacity to capture relevant interactions. A summary of different types of protein–ligand interaction fingerprints is listed in Table 1. A list of currently available software for calculating interaction fingerprints is shown in Table 2.

## 3. Case Study of Structural Interaction Fingerprint Application

**Figure 3:**
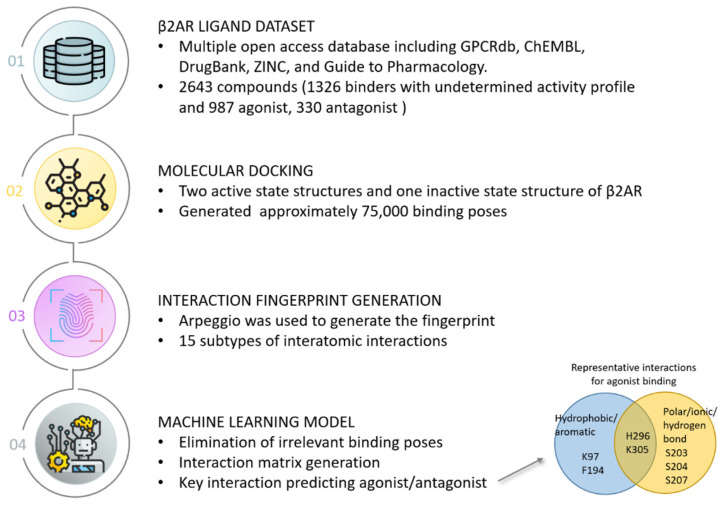
Case study of Jimenez-Roses et al. [53]. Workflow of utilizing interaction fingerprints extracted from docking poses as input for machine learning model to identify key residues for ligand pharmacological activity on β2 receptors.

In this section, we highlight two case studies that incorporated structural interaction fingerprints into machine learning. The first case study demonstrated that molecular docking and machine learning can be combined to reveal key structure–activity relationships for drug targets [53]. The researchers compiled a dataset of approximately 2700 known ligands for the β2 adrenoceptor (β2AR). They computationally docked these ligands to β2AR structures to generate approximately 75,000 poses and calculated atomic interaction fingerprints describing receptor–ligand interactions. Machine learning models were trained on these fingerprints to predict whether ligands act as agonists or antagonists. Figure 3 shows the detailed workflow of this work. The models identified specific hydrophobic and polar contacts with receptor residues that differentiate agonists and antagonists. Agonists were found to preferentially interact with residues K97, F194, S203, S204, S207, H296, and K305 while antagonists were found to favor residues W286 and Y316. This structure–activity relationship modeling approach achieved high accuracy in predicting ligand pharmacological activity and provided molecular insights into β2AR activation and inhibition. This study demonstrates the power of interaction fingerprint-driven machine learning for elucidating ligand binding mechanisms and guiding rational drug design. The results from this case study revealed that structural interaction fingerprints derived from docking poses offer insights into the environment surrounding the ligand, which can be useful for differentiating the potential biological activities of ligands.

**Figure 4:**
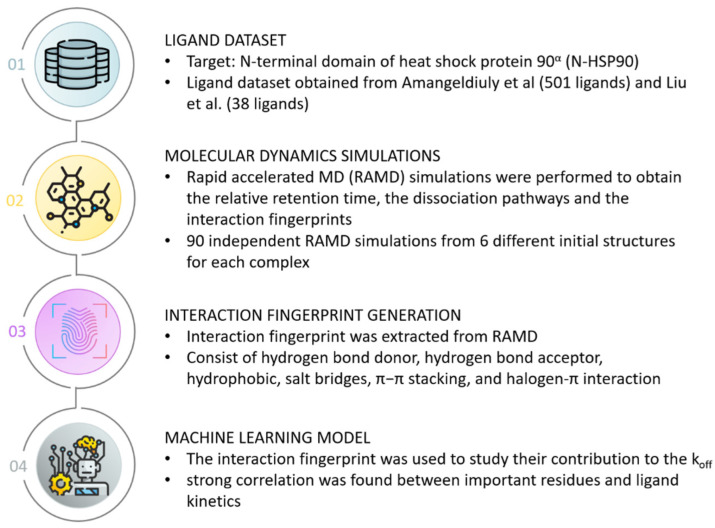
Case study of Zhou et al. [54]. Workflow of utilizing interaction fingerprints extracted from MD simulations as input for machine learning model to identify correlation between key residues and ligand kinetics. The ligand dataset in this case study was obtained from Amangeldiuly et al. [55] and Liu et al. [56].

The second case study introduced a machine learning strategy employing an innovative molecular representation termed RPM (retrosynthesis-based pre-trained molecular) representation to predict protein–ligand dissociation rates (k_off_) [54]. The RPM representation was constructed through training on retrosynthesis reaction data, enabling the encapsulation of molecular reactivity and functional group information. Subsequently, these RPM features were fed into a partial least squares regression model to predict the k_off_ values for 501 inhibitors spanning 55 proteins. Impressively, the RPM-based model demonstrated superior performance compared to other pre-trained representations such as the molecular pre-training graph-based deep learning framework and geometry-enhanced molecular representation, achieving a noteworthy Pearson correlation coefficient of 0.76 on this specific dataset. To exemplify its application, the model was further evaluated using 38 novel inhibitors targeting the N-terminal domain of the heat shock protein 90α (HSP90), yielding a commendable correlation of 0.73 with experimental k_off_ values. In-depth mechanistic insights into the kinetics were sought through accelerated molecular dynamics simulations, which obtained data on relative retention times and protein–ligand IFPs along the dissociation trajectory. Figure 4 illustrates the detailed workflow of this case study. The simulated k_off_ values exhibited reasonable agreement with experimental results, with the IFPs elucidating important residues like N51, S52, and L107 that significantly influence the dissociation process. In an additional validation, the machine learning model coupling with molecular dynamics simulation was extended to two new HSP90 inhibitors absent from the training set. Encouragingly, the model accurately predicted their relative k_off_ values, which were aligned with experimental observations. Furthermore, the IFP analysis offered detailed insights into how substituents modulated binding kinetics. This case study combined different approaches and offered a comprehensive exploration of the molecular attributes and interactions that govern binding kinetics, thereby underlining its potential utility for kinetics-focused drug design endeavors.

## 4. Future Perspective

Molecular fingerprints have become indispensable cornerstones in the realm of computational drug discovery, offering informative representations of ligands for property prediction and activity modeling. In this landscape, the realm of molecular fingerprints stands at an exciting crossroads, with 2D fingerprints providing simplicity and ease of use, while 3D structural interaction fingerprints hold the tantalizing potential to intricately encapsulate the minutiae of interactions within protein–ligand complexes. The future trajectory of this field is poised for further advancement, driven by the synergy of hybrid fingerprint design and technological progress. The amalgamation of 3D structural interaction descriptors with other properties, such as physicochemical attributes, has the potential to elevate the accuracy of ligand bioactivity predictions. By encompassing both structural intricacies and physicochemical subtleties, hybrid fingerprints extend the horizons of molecular characterization, and the application of advanced machine learning techniques holds the key to their optimal integration. As computational methodologies advance and resources expand, the landscape for harnessing the potential of 3D fingerprints in drug discovery grows even more fertile. The interplay of refined machine learning algorithms, augmented structural datasets, and enhanced computational power opens new possibilities and opportunities in interaction fingerprint design, training, and prediction, with deep learning strategies poised to unveil profound insights from intricate 3D interaction patterns.

Yet, as the future of molecular fingerprints shines brightly, it is not without its challenges. One such limitation lies in the dependency of 3D fingerprints on the accessibility of protein–ligand complex structures. Nonetheless, the ongoing advancements in structural determination techniques contribute to an increasing abundance of structures, facilitating the progress of molecular fingerprint development. Another drawback is the insufficient incorporation of the energy terms necessary to comprehensively characterize the interactions occurring between proteins and ligands. Recent deep learning-based scoring functions may potentially solve this problem. Decoding complex machine learning models constructed on 3D fingerprints is another challenge. The process of unraveling the pivotal interacting features driving a model’s predictions remains an active area of exploration. Novel strategies are essential to deconstruct model outputs into interpretable interaction insights, which in turn can illuminate pathways for molecular optimization. Moreover, the intricacy of binding site plasticity and induced fit effects introduces complexities in accurately characterizing interactions solely from static structural data. Another limitation is the reliance on the availability of known ligand–protein interaction information. In both case studies, the target has a large number of known ligands that can be used for model training. However, for targets that have few or no known ligands, for which the discovery of new ligands is in higher demand, this method would not be as applicable.

Overall, interaction fingerprints hold immense promise but require continued research to fully harness their potential and overcome existing limitations, unlocking new vistas of discovery and application.

## Figures and Tables

**Table 1:** Different types of protein–ligand interaction fingerprints and their characteristics.

Types of Protein–Ligand Interaction Fingerprints	Characteristics and Pattern Types	Length	Reference
Structural IFP	Uses well-defined interaction types such as hydrogen bond, halogen bonds, and π-π stacking	Each residue is represented by a seven-bit long bit string	[33,34]
Python-based protein–ligand interaction fingerprint (PyPLIF)	Uses well-defined interaction types such as hydrogen bond, halogen bonds, and π-π stacking	Seven bits represent seven different interactions for each residue	[39]
Triplet IFP	Uses two interacting atoms and an interaction pseudoatom positioned at three potential locations: the geometric center of the interacting atoms, the interacting protein atom, and the interacting ligand atom to encode different interaction types (7 types) at defined distance ranges (6 ranges)	210 integers	[30]
Atom-pairs-based interaction fingerprint (APIF)	Considers the relative positions of the atom pairs instead of the absolute locations of the individual interactions	294 bits	[40]
Simple ligand–receptor interaction descriptor (SILIRID)	Groups interactions by residue type, the interactions included are hydrophobic, aromatic face to face, aromatic edge to face, H-bond donated by the protein, H-bond donated by the ligand, ionic bond with protein cation and protein anion, and interaction with metal ion	168 integers (corresponds to the product of 20 amino acids and 1 co-factor and 8 interaction types per amino acid)	[41]
Structural protein–ligand interaction fingerprint (SPLIF)	Encodes interacting ligand and protein fragments by representing them as circular fingerprints using Extended Connectivity Fingerprints (ECFP2) and generates integer identifiers to represent each substructure fragment	Length depends on the number of interacting fragments identified	[42]
Protein–ligand extended connectivity fingerprint (PLECFP)	Pairs and hashes the ECFP environment from the interacting ligand and protein atoms to represent contacts and interactions between the molecules	The raw folded fingerprint consists of integers between 0 and 2^32^ (32 bits)	[43]

**Table 2:** Available software for calculating structural interaction fingerprints.

Software/Web Server	Types of Input Complex	Input Format	MD Trajectory Analysis	Reference
Arpeggio	All combinations between ligand, protein, DNA and RNA molecules	PDB	N/A	[44]
fingeRNAt	All combinations between ligand, protein, DNA and RNA molecules	PDB and SDF	N/A	[45]
getContacts	All combinations between ligand, protein, DNA and RNA molecules	VMD	N/A	getcontacts.github.io (accessed on 2 November 2023)
Ichem	Protein ligand complex only	Mol2	N/A	[37]
LUNA	Protein ligand and protein–protein complex	PDB, Mol, Mol2, and RDKit	N/A	[46]
MD-IFP	Ligand protein complex only	MDAnalysis	Yes	[47]
ODDT	Ligand protein complex only	OpenBabel and RDKit	N/A	[48]
PLIP	All combinations between ligand, protein, DNA and RNA molecules	PDB	N/A	[49]
ProLIF	All combinations between ligand, protein, DNA and RNA molecules	MDAnalysis and RDKit	Yes	[50]
PyPLIF HIPPOS	Ligand protein complex only	PDBQT and Mol2	N/A	[39]
Schrodinger	Ligand protein complex only	SDF, PDB, and MAE	N/A	[51,52]
